# A rare case of laparoscopy towards SLE with lupus mesenteric vasculitis induced ascites

**DOI:** 10.1186/s12893-019-0533-5

**Published:** 2019-07-02

**Authors:** Kamleshsingh Shadhu, Dadhija Ramlagun, Xiaochun Ping

**Affiliations:** 1 Department of General Surgery, The First Affiliated Hospital of Nanjing Medical University, Guangzhou Road, 300, Gulou District, Nanjing, Jiangsu 210029 People’s Republic of China; 2 Department of Gastric Surgery, The First Affiliated Hospital of Nanjing Medical University, Guangzhou Road, 300, Gulou District, Nanjing, Jiangsu 210029 People’s Republic of China

**Keywords:** Systematic lupus erythematosus (SLE), Laparoscopy, Acute abdomen

## Abstract

**Background:**

Diagnosis and management of acute abdomen secondary to systematic lupus erythematosus (SLE) has always been a clinical challenge.

**Case presentation:**

A 21-year-old lady, with BMI 17.7, presented to our department with acute abdomen. Laparoscopy was carried out to exclude surgical emergency when conservative regimen failed. The patient revealed a history of purpuric changes and lupus test was positive for SLE.

**Conclusion:**

Based on our experience, early laparoscopy to alleviate acute abdomen has shown to improve the prognosis of the patient.

## Background

Systematic lupus erythematosus (SLE) is a chronic inflammatory disease. The extent of gastrointestinal tract involvement in patients with SLE is rare and the outcomes remain unclear. A retrospective single-center analysis reports an incidence of 0.6% of gastrointestinal complications in patients with SLE [1]. The use of early surgical intervention instead of surgical therapy as an optimum treatment for lupus mesenteric vasculitis (LMV) is controversial [2–4]. We here in report a case of a young lady who had abdominal ascites via SLE with LMV.

## Case presentation

**Fig. 1:**
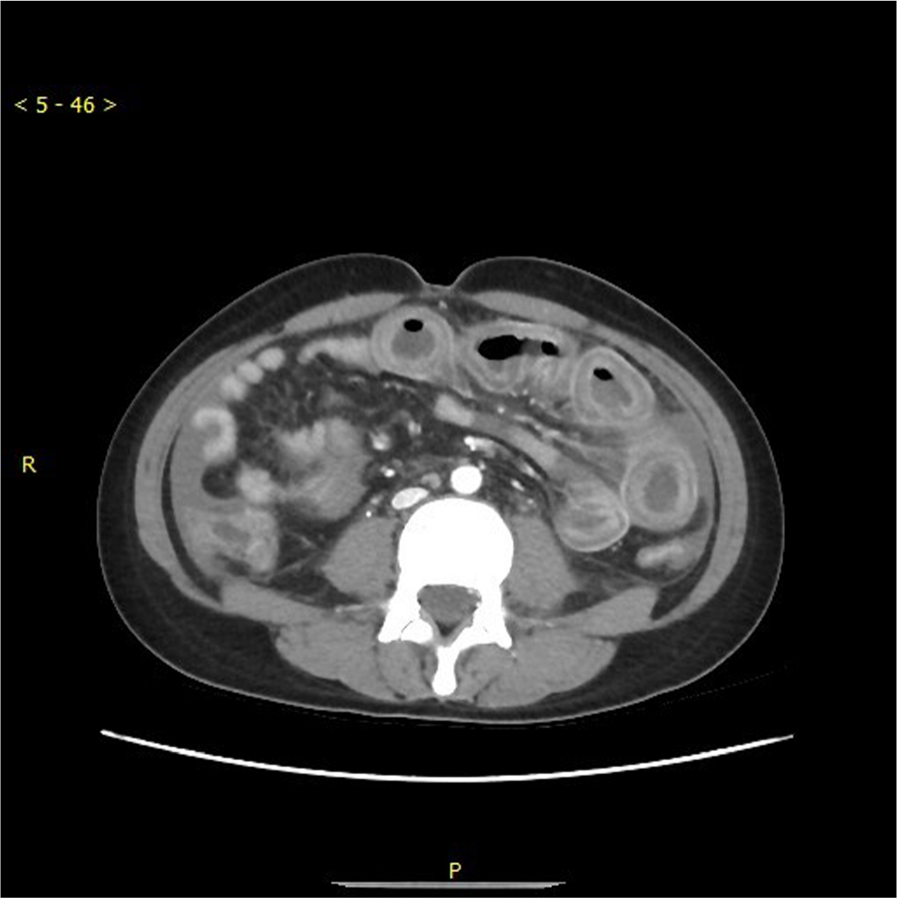
Transverse section CT scan showing dilatation of small intestine and ascites

**Fig. 2:**
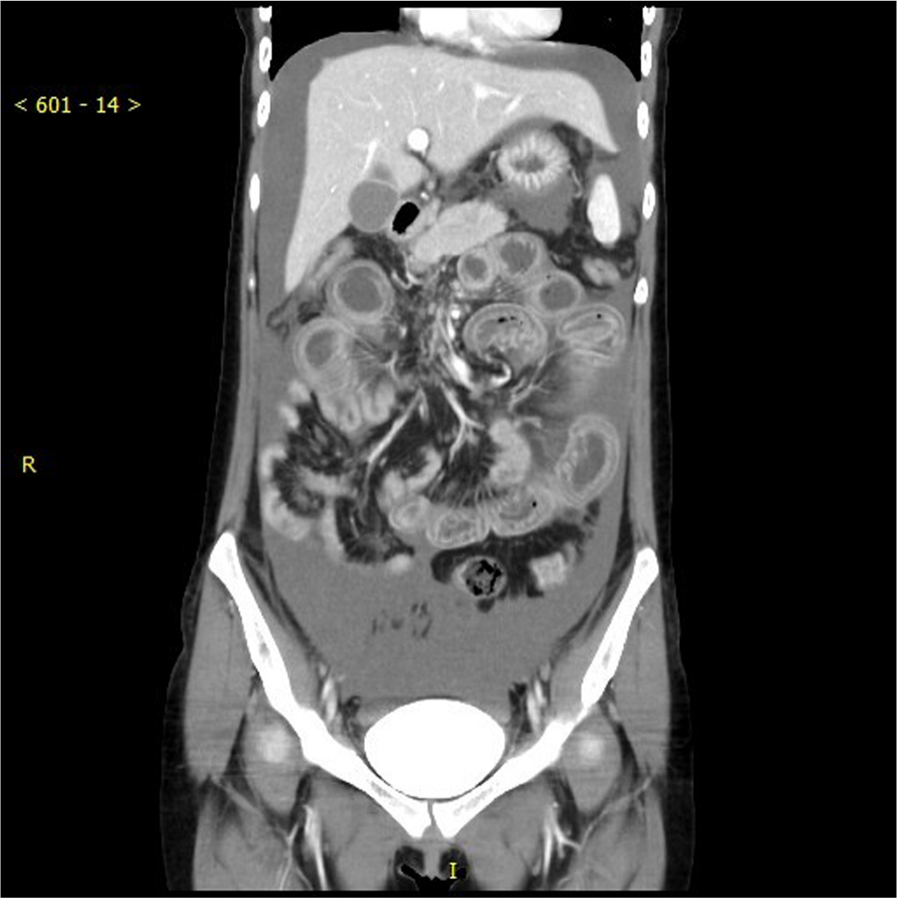
Coronary section of CT scan showing dilatation of proximal small intestine, massive ascites, no occlusion or filling defects in major mesenteric vessels

**Fig. 3:**
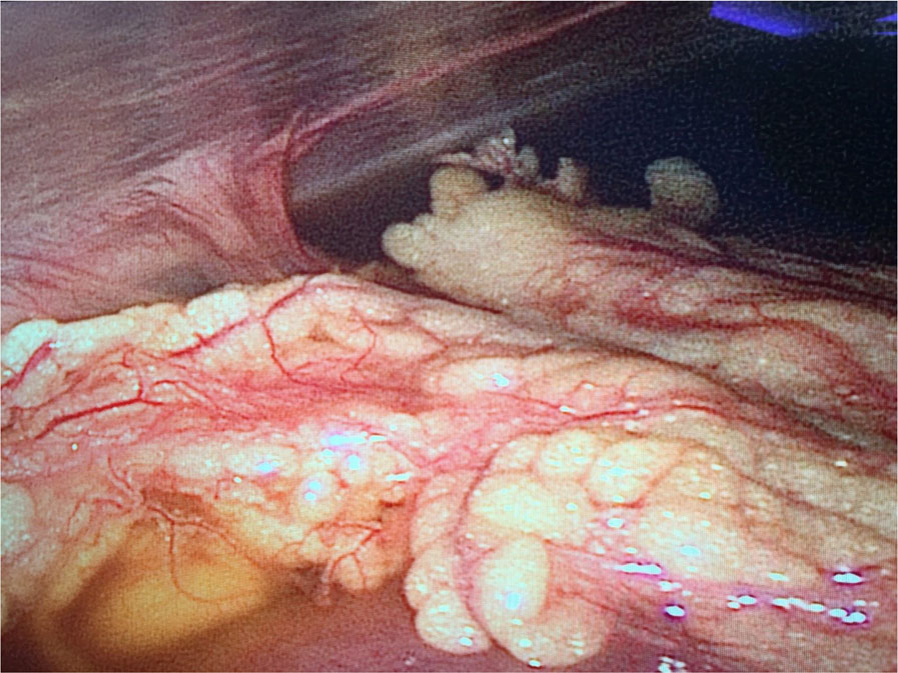
Intraoperative picture showing massive yellowish ascites

**Fig. 4:**
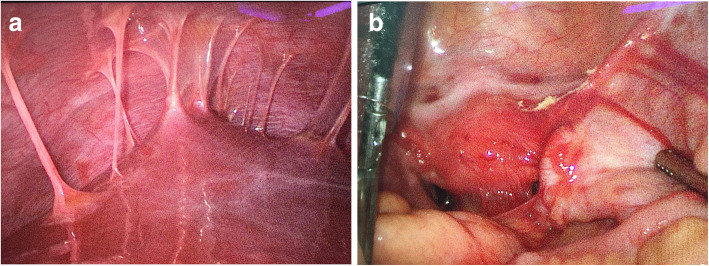
**a** Intraoperative picture showing adhesive bands (violin-string) between the liver and the diaphragm. **b** Intraoperative picture showing adhesions in the pelvic cavity

**Fig. 5:**
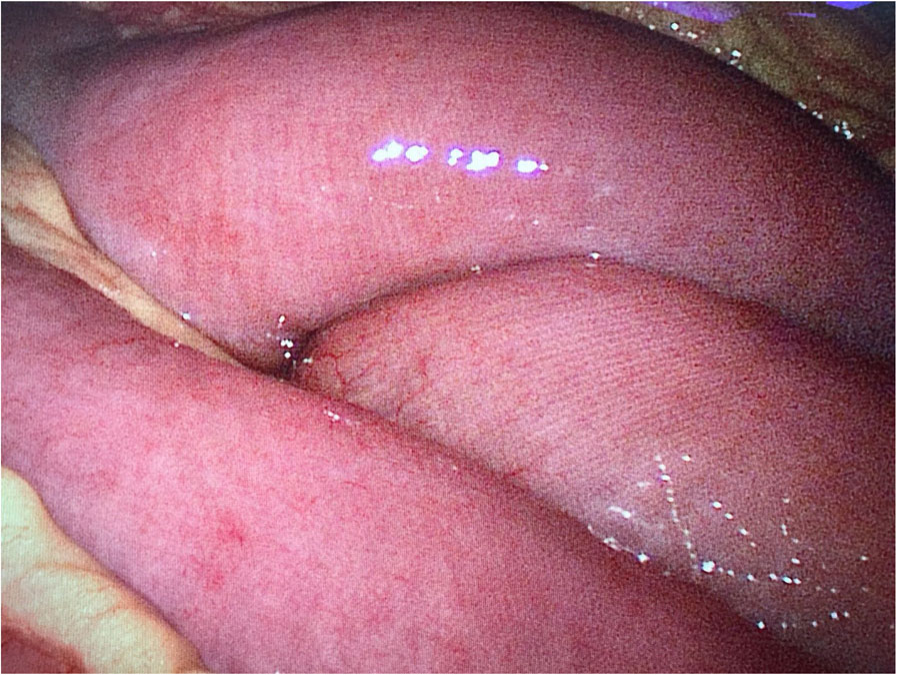
Intraoperative picture showing enlarged diameter of the proximal jejunum

A 21 years old female patient, of body mass 47 kg and BMI 17.7, came to our emergency department due to paroxysmal abdominal pain for 4 days. The pain was intermittent, moderate to severe, cramping in the epigastric area. She also had diarrhoea 2 days ago and hadn’t had any bowel movements ever since. A similar episode occurred 2 months ago which subsided spontaneously over few days. However, the pain she had this time was so severe that medical treatments she received in urgent clinic, at another hospital, couldn’t provide relief. Upon arriving at the emergency room of our hospital, her vital signs were 37.2 °C, heart rate 98 bpm, respiratory rate 18 bpm, blood pressure 126/92 mmHg. Physical examination revealed a moderately distended abdomen, tenderness in the epigastric area without rebound, positive shifting dullness, and hypoactive bowel sounds. Laboratory tests found white blood cells count was 17.2*10^9/L, neutrophils 88.7% and D-dimer 11.7 mg/L. Abdominal CT scan showed dilatation of proximal small intestine with thickened walls and air-fluid levels and accumulation of massive abdominal ascites. There was no sign of occlusion or filling defect in the superior mesenteric artery and vein, or their distal branches (Figs. 1 & 2). She denies any past medical history or on any medications. She is sexually active and had her immunization up to date. Due to the worsening nature of her pain after conservative treatments, acute abdomen was suspected, and a diagnostic laparoscopy was performed to exclude any surgical emergencies. During the surgery, 2500 mL of yellowish ascites were drained (Fig. 3). Multiple adhesive bands were seen between the liver and the diaphragm, and in the pelvic cavity (Figs. 4a & 4b). Part of omentum was adhered to the right side of the pelvic floor, which was lysed. Inspection of the whole length of small intestine showed dilation and thickened walls of the jejunum, 50 cm in length (Fig. 5). The ileum was normal, and no obstruction point was found. The colour and peristalsis of the intestines were normal. The patient was diagnosed as idiopathic peritonitis and pseudo-ileus intra-operatively. The characteristics of the drained ascites was shown in Table 1. Supportive treatments as well as antibiotics, including cefoperazone and metronidazole, were given immediately after the surgery as Fitz-Hugh-Curtis syndrome was also suspected. However, on the morning of postoperative day (POD) 1, another 2950 mL of ascites were found in the drainage tube. The patient was haemodynamically unstable. Aggressive resuscitation was initiated. The family of the patient later revealed that 6 months ago she had multiple erythema on her palms and cheek, which were purpuric like changes and subsided after herbal medicine. The lupus mesenteric vasculitis (LMV) was then suspected. The lupus tests together with other diagnostic tests were carried out. Their results were shown in Tables 2 and 3. She was positive for anti-nuclear antibody (ANA), anti-Smith, anti-u1-snRNP, anti-Ro, anti-dsDNA antibodies and low in complements C3 and C4. The patient was diagnosed with systematic lupus erythematosus (SLE) with lupus mesenteric vasculitis. She was treated with 80 mg IV methylprednisolone per day and 0.2 g of oral hydroxychloroquine twice a day with rapid improvement of abdominal symptoms. She resumed normal diet few days after her ascites diminished and was discharged on POD 12. On follow-up, the patient continued her treatments at the rheumatology department and had no surgical associated complications.

**Table 1:** Laboratory findings of ascites

Variables	Results
Appearance	Yellow
Transparency	Transparent
Rivalta test	Postive
Protein (g/L)	30.2
Albumin (g/L)	12.3
Amylase (IU/L)	< 30
Glucose (mmol/L)	5.37
LDH (U/L)	978
Effusion-serum protein proportion	0.92
Effusion-serum LDH proportion	7.13
Serum-ascites albumin gradient (SAAG) (g/L)	6.2
Nucleated cell count (per uL)	401
Cytology	Neutrophils (60.4%), lymphocytes and mesothelial cells, but no tumor cells
Tuberculosis smear	Negative
Bacterial and tuberculosis culture	Negative

**Table 2:** Laboratory tests of blood count, liver and renal function and tumour markers

Variables	Values	References
WBC (10^9^/L)	16.84	3.5–9.5
Hb (g/dL)	10.1	11.5–15.5
Platelet (10^9^/L)	202	125–350
C-reactive protein (mg/L)	10.7	0.0–8.0
ESR (mm/h)	6	< 26
Cr (umol/L)	44.1	44.0–133.0
BUN (mmol/L)	5.28	2.9–8.2
Total protein (g/L)	32.7	65.0–85.0
Albumin (g/L)	18.5	40.0–55.0
Urinalysis: protein/blood	+/−	–
Urine protein (mg/L)	408	0–100
24 h urine protein (mg)	1428	24–141
AFP (ng/mL)	1.88	< 20.00
CEA (ng/mL)	0.54	< 4.70
CA-125 (U/mL)	38.4	< 35.0
CA-199 (U/mL)	7.35	< 16.30
NSE (ng/mL)	10.56	< 16.30

**Table 3:** Laboratory tests of autoimmune diseases

Variables	Results	References
Anti-nuclear antibody, titer	Positive, 1:3200	–
A-β2-GP1 (RU/mL)	3.4	< 20.0
A-dsDNA-3 (IU/mL)	80.1	< 100
Anti-cardiolipin IgG (U)	5.7	< 20.0
Anti-U1-snRNP	Positive	–
Anti-SmD1	Positive	–
Anti-RPP	–	–
Anti-nucleosome	–	–
Anti-histone	–	–
Anti-SSA/Ro52	Positive	–
Anti-SSA/Ro60	Positive	–
Anti-SSB-2e	–	–
Anti-Scl-70	–	–
Anti-CenpB	–	–
Anti-Jo-1	–	–
Anti-Mi-2	–	–
Anti-PM-Scl	–	–
Anti-Ku	–	–
Anti-PCNA	–	–
AMA-M2–2	–	–
Anti-dsDNA	Weakly positive	–
IgG (g/L)	14.5	7.00–16.00
IgA (g/L)	1.7	0.70–4.00
IgM (g/L)	1.12	0.400–2.300
C3 (g/L)	0.2	0.9–1.8
C4 (g/L)	< 0.064	0.100–0.400

## Discussion and conclusion

It is observed that in 87% of patients with SLE, involving gastrointestinal tract, abdominal pain was almost a constant sign [5]. Besides, it has been shown, by Medina et al. [6], that adults with SLE, who had intrabdominal vasculitis or thrombosis, had higher systematic lupus erythematosus disease activity index (SLEDAI) scores and were associated with surgical abdomen. Since 1993 the SLEDAI has been used as an index of disease activity and the score for our case was 8 [7]. This was calculated via the features of proteinuria (4), malar rash (2) and hypocomplementemia (2) which were present in the lady [7]. In a study the aetiologies of abdominal pain have been compared in patients with active and inactive SLE in which patients with active disease (SLEDAI score > 5) have a higher prevalence of LMV than those with inactive disease. Therefore, this showed that mesenteric vasculitis can be combined with thrombocytopenia, central nervous system involvement, cutaneous vasculitis and lymphopenia [6].

Besides, a case series reported by Kishimoto et al. [8] showed that patients with acute gastrointestinal distress syndrome have recurrent lupus enteritis based on mesenteric vasculopathy characterised by reversible intestinal wall oedema accompanied by significant hypocomplementemia. Another study showed that anti-proliferative cell nuclear antigen antibody was found to be more frequently detected than in lupus patients without pseudo-ileus [9]. SLE-reported pseudo-ileus patients had higher frequency of positive anti-Ro and anti-RNP antibodies compared to lupus patients without pseudo-ileus, was reported by Mok et al. [10].

Moreover, based on literatures, ascites occurs in 8–11% of adult patients with SLE [11]. The course of differentiating a patient with an acute abdomen secondary to SLE as the sole presenting symptom is a real challenge. For our case, the dilatation of jejunum, presence of massive ascites, elevated white blood cell count and failed conservative regimen to alleviate the severe abdominal pain prompted us to perform a laparoscopy to exclude any surgical emergency. Common CT findings in patients with LMV include dilated bowel, focal or diffuse bowel-wall thickening, abnormal bowel-wall enhancement (a double halo or target sign), mesenteric oedema, engorgement of mesenteric vessels (comb sign) and ascites [12–15]. These signs are also shared by disorders such as mechanical bowel obstruction and inflammatory bowel disease, which can limit the accuracy of the diagnosis. Nevertheless, CT findings can help to differentiate LMV from thrombo embolic disease [16]. Literatures have reported that surgical intervention improves prognosis of patients who fail to respond to conservative management [6].

In addition, Fitz-Hugh-Curtis syndrome was initially suspected. In 1930, Curtis [17] reported the violin-string appearance between the anterior hepatic surface and abdominal wall. This was also observed in our patient. However, the patient revealed that she had her first sexual intercourse 2 months ago, using condom as barrier method. The bacterial culture was negative and there was no sign of tuberculosis. Laparoscopy has been postulated as the gold standard for diagnosing Fitz-Hugh-Curtis syndrome [18].

We, therefore, conclude that diagnosing a patient with acute abdomen secondary to SLE remains a clinical challenge. Surgical intervention helps to alleviate the acute abdomen and exclude any surgical emergency when conservative approach fails. Nevertheless, this is a single case study and conclusion may only be drawn from our experience.

## Data Availability

Not applicaple

## Abbreviations

CT: Computer tomography ANA Anti-nuclear antibody
LMV: Lupus mesenteric vasculitis
POD: Post-operative day
SLE: Systematic lupus erythematosus
SLEDAI: Systematic lupus erythematosus disease activity index
